# Changes in practice patterns in Japan from before to after JSDT 2013 guidelines on hemodialysis prescriptions: results from the JDOPPS

**DOI:** 10.1186/s12882-021-02543-3

**Published:** 2021-10-14

**Authors:** Tadashi Tomo, Maria Larkina, Ayumi Shintani, Tomonari Ogawa, Bruce M. Robinson, Brian Bieber, Lisa Henn, Ronald L. Pisoni

**Affiliations:** 1 Clinical Engineering Research Center, Oita University, 5593 Idai-gaoka,1-1, Hasama-machi, Yufu-City, Oita Japan; 2 Arbor Research Collaborative for Health, Ann Arbor, USA; 3 Currently at Michigan Medicine, Department of Internal Medicine, Nephrology Division, University of Michigan, Ann Arbor, Michigan USA; 4 Department of Medical Statistics, Graduate School of Medicine Osaka City University, Osaka, Japan; 5 Department of Nephrology and Blood Purification Center Saitama Medical Center, Medical University, Saitama, Japan

**Keywords:** Hemodialysis, guidelines, dialysis adequacy, Kt/V, hemodiafiltration, blood flow rate

## Abstract

**Background:**

The Japanese Society for Dialysis Therapy (JSDT) published in 2013 inaugural hemodialysis (HD) guidelines. Specific targets include 1.4 for single-pool Kt/V (spKt/V) with a minimum dose of 1.2, minimum dialysis session length of 4 hours, minimum blood flow rate (BFR) of 200 mL/min, fluid removal rate no more than 15 mL/kg/hr, and hemodiafiltration (HDF) therapy for certain identified symptoms. We evaluated the effect of these guidelines on actual practice in the years spanning 2005 – 2018.

**Methods:**

Analyses were carried out to describe trends in the above HD prescription practices from December 2005 to April 2013 (*before guideline publication*) to August 2018 based on prevalent patient cross-sections from approximately 60 randomly selected HD facilities participating in the Japan Dialysis Outcomes and Practice Patterns Study.

**Results:**

From April 2006 to August 2017 continual rises occurred in mean spKt/V (from 1.35 to 1.49), and percent of patients having spKt/V>1.2 (71% to 85%). Mean BFR increased with time from 198.3 mL/min (April 2006) to 218.4 mL/min (August 2017) , along with percent of patients with BFR >200 ml/min (65% to 85%). HDF use increased slightly from 6% (April 2006 and August 2009) to 8% by April 2013, but increased greatly thereafter to 23% by August 2017. In contrast, mean HD treatment time showed little change from 2006-2017, whereas mean UFR declined from 11.3 in 2006 to 8.4 mL/Kg/hour in 2017.

**Conclusions:**

From 2006 – 2018 Japanese HD patients experienced marked improvement in reaching the spKt/V target specified by the 2013 JSDT guidelines. This may have been due to moderate increase in mean BFR even though mean HD session length did not change much. In addition, HDF use increased dramatically in this time period. Other HD delivery changes during this time, such as increased use of super high flux dialyzers, also merit study. While we cannot definitively conclude a causal relationship between the publication of the guidelines and the subsequent practice changes in Japan, those changes moved practice closer to the recommendations of the guidelines.

## Background

The National Cooperative Dialysis Study, an observational study, reported an association between dialysis dose and morbidity/mortality in hemodialysis (HD) patients [1]. Subsequently, several observational studies showed that higher dialysis dose and longer dialysis treatment time were associated with lower mortality rates. These studies confirmed that survival of HD patients is greatly influenced by dialysis prescription [2–5].

Based on these findings, the Kidney Disease Outcomes Quality Initiative (KDOQI) guidelines for hemodialysis adequacy were developed and recommended that single pool Kt/V should be 1.2 or higher (excluding residual kidney function), as a measure of dialysis dose for thrice weekly HD [6]. However, in the randomized clinical HEMO Study in 2002, higher dose hemodialysis, yielding an average single-pool Kt/V of 1.71, showed no survival benefit over lower dose hemodialysis at a single-pool Kt/V of 1.32 [HR= 0.96; 95% C.I. (0.84,1.10)] [7]. Nevertheless, subgroup analysis suggested that survival was increased for women in the higher dose Kt/V group [7, 8]. Subsequently, Port et al [9], using a much larger observational study dataset, similarly found increased survival for women at higher dose Kt/V levels. It should be noted that the hemodialysis treatment time in the HEMO Study was shorter and that the dialysis prescriptions in the HEMO Study were quite different from those in Japan and Europe. Therefore, it is difficult to apply these results to patients in Japan and Europe. Nonetheless, Kimata et al [10] based on Japan-DOPPS data from 1999-2011, observed lower Kt/V to be associated with elevated mortality among Japanese HD patients and more so among women (HR = 1.13 per 0.1 lower Kt/V, 95% CI: 1.07–1.20) than among men (HR = 1.06 per 0.1 lower Kt/V, 95% CI: 1.00–1.12).

Hemodialysis prescription varies greatly among countries. Considerable diversity in dialysis prescription is also reported in the Dialysis Outcomes and Practice Patterns Study (DOPPS) [11]. In Japan, dialysis patients typically receive a lower dialysis dose, with a dialysis treatment time of 4 hours and a blood flow rate of approximately 200 mL/min [12]. It is important to consider that mortality of dialysis patients is greatly affected not only by dialysis dose but also by dialysis prescription factors including dialysis treatment time, blood flow rate, ultrafiltration rate, type of hemodialysis filter, and treatment modality [13]. In May 2013, the guidelines for hemodialysis prescription were published by the Japanese Society for Dialysis Therapy. The guidelines provide recommendations on dialysis-related factors including dose, treatment time, blood flow rate, and fluid removal rate. The recommendations are as follows: the minimum dialysis dose of single-pool Kt/V is 1.2 and the target dose is 1.4; the minimum blood flow rate is 200 mL/min; the minimum treatment time is 4 hours; and the fluid removal rate is 15 mL/kg/hr or lower. In addition, hemodiafiltration (HDF) is recommended for enhancing the removal of middle molecular-weight solutes (e.g., Beta-2-Microglobulin), for reducing inflammatory cytokines, and for alleviating patient symptoms such as poor appetite, itching, joint pain, and fatigue [14].

In this study, we investigated the impact of the 2013 Japanese Society for Dialysis Therapy Clinical Guideline for “Maintenance Hemodialysis: Hemodialysis Prescriptions” (hereafter referred to as the Guidelines) on hemodialysis prescription in Japan using the data of the Japan-DOPPS from 2005–2018 (DOPPS phase 3, 4, 5, and 6).

## Methods

### Data source

The DOPPS (http://www.dopps.org) is an international prospective cohort study of in-center HD patients randomly selected from a representative sample of dialysis facilities [11, 12, 15]. This analysis included data from 3,491 HD patients participating in Japan (JDOPPS) phases 3 (2005–2008), 4 (2009–2011), 5 (2012–2015), and 6 (2016-2018), for a total follow up time of 7,087 patient years. Data were reported based on patient medical records, using demographic variables and comorbidity reported at study entry in each study phase. In contrast, laboratory measurement values and medication prescriptions were based on data reported at study entry and updated every 4 months during study participation. All variables were collected using the same data collection tools for all JDOPPS participants. Study approval was obtained by a central institutional review board.

### Data Analysis

Our analyses compared the achievement of dialysis adequacy guideline measures between two periods: before and after the May 2013 publication of the JSDT guidelines (GL). The studied GL measures were: single pool Kt/V (spKt/V), blood flow rate (BFR), HD treatment time (TT), use of hemodiafiltration (HDF), and ultrafiltration rate (UFR).

Multivariable linear and generalized linear mixed models were used to assess the slope of the monthly changes in each studied GL measure (i.e., rate of change) that occurred before and after the May 2013 JSDT guideline publication. These models were adjusted for potential confounders including age, sex, time on dialysis, use of HDF (except when HDF use was the outcome), and 13 comorbid factors (diabetes mellitus, cerebrovascular accident (CVA), congestive heart failure, peripheral vascular disease, coronary artery disease, hypertension, other cardiovascular (CV) diseases, recurrent cellulitis/gangrene, cancer other than skin, gastrointestinal (GI) bleeding, lung disease, neurologic disease, and psychiatric disorder). Auto-regressive, order 1 covariance structure was used based on assessment of best model fit as assessed by the Akaike information criterion (AIC) [16] and Bayesian information criterion (BIC) [17], also known as the Schwarz information criterion, among structures that converged across all analyses using multivariable linear mixed models. For binary outcome variables, multivariable generalized estimating equation (GEE) regression models were applied in a manner similar to the above. Subgroup analyses were performed on the following samples stratified by: sex, age (less than vs ≥70 years old), and blood flow rate (less than vs. ≥200 mL/min). Standard descriptive statistics characterized the DOPPS patients included in the study at several cross sections, representing each study phase, during follow up (April 2006, August 2009, April 2013, and August 2017).

Any longitudinal patient measures collected during the 13 years of the study period were included in the slope models. Median (interquartile range) of patient follow up was 3.76 (2.59, 6.24) years and median number of records was 12 (9, 20). Median (IQR) number of outcome variables of interest was 12 (8, 19) for BFR, TT, and HDF, and 11 (7, 19) for Kt/V, and UFR. Adjustment variables (age, time since start of dialysis, use of HDF, and 13 comorbidities) were updated at the beginning of each study phase. The effect of time was assessed with a variable indicating months counting to or from the GP (May 2013), an indicator of period prior or post GP, and a cross-product term of the two. The model for the outcome of use of HDF was necessarily unadjusted for HDF. However, reimbursement for HDF changed in April 2012, and its use likely changed in response to this. Consequently, we included in the HDF outcome model an indicator variable representing observations before vs. after April 2012 to capture this external effect.

All analyses used SAS software, version 9.4 (SAS institute, Cary, NC).

## Results

**Figure 1:**
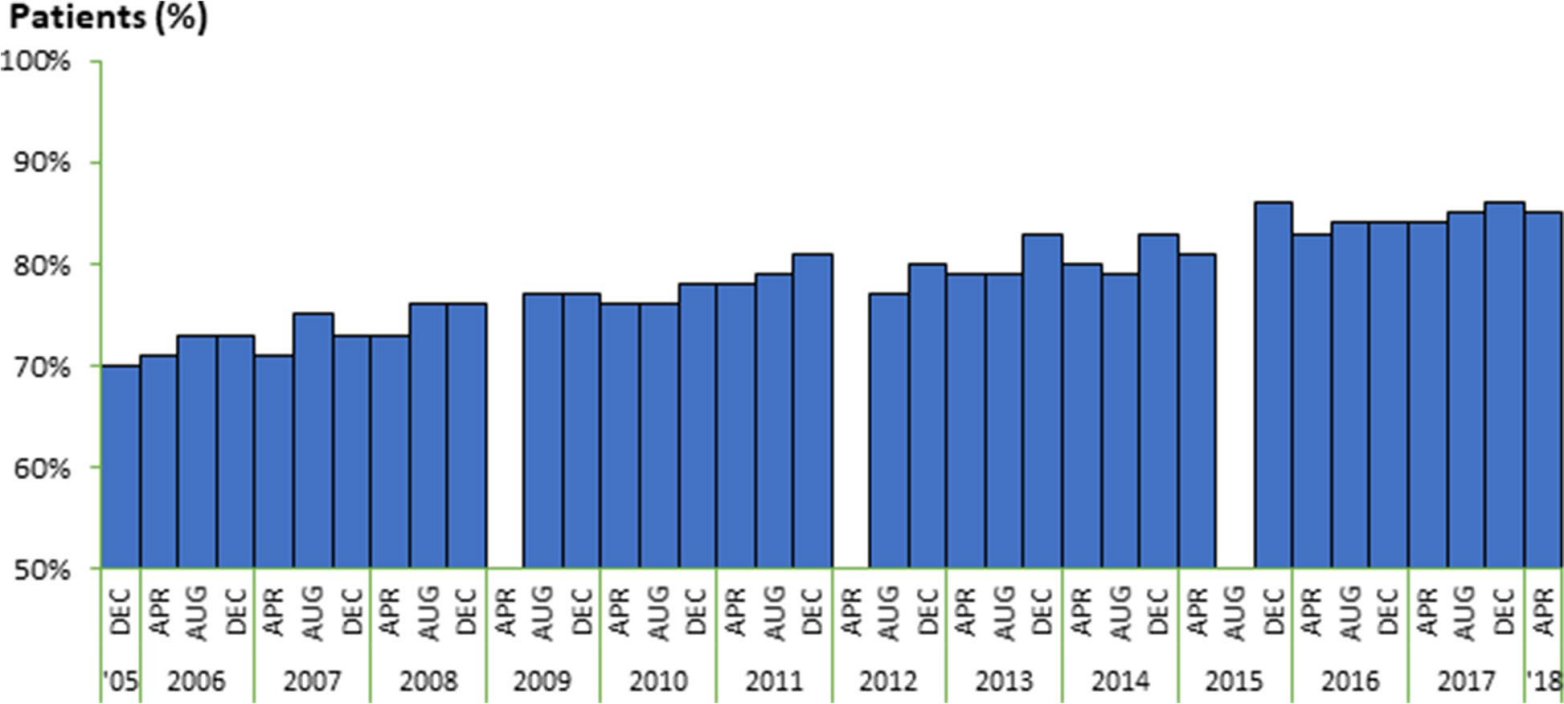
Single-pool Kt/V ≥1.2

Table 1 shows the changes before (April 2006, August 2009, April 2013) and after (August 2017) publication of the JSDT Guidelines for dialysis adequacy. The mean spKt/V increased with time, from 1.35 (April 2006) to 1.40 (August 2009) and to 1.42 (April 2013), even before publication of the Guidelines. The value further increased to 1.49 after publication of the Guidelines (August 2017). The pre-publication slope for mean spKt/v was +0.01481/month, and the post-publication slope for mean spKt/v was +0.01781/year, with this slope difference of 0.003/year not being statistically significant(95% C.I. [−0.0022, 0.0082], Table 2). The percentage of patients having a single pool Kt/V of ≥1.2 also increased with time from 71% in April 2006, to 77% in August 2009 and to 79% by April 2013 before publication of the Guidelines, while further increasing to 85% after publication of the Guidelines (Figure 1). In case-mix adjusted logistic regression mixed models of the odds of patients having spKt/V ≥1.2 vs. not over time, the pre-publication slope was +0.109 units/year. By contrast, the post-publication slope was +0.1292 units/year, resulting in a slope difference of 0.02015 units/year (95% C.I. [−0.0434, 0.0837], Table 3). In a sensitivity analysis, when models were not adjusted for HDF use, the post-/pre-slope difference was seen to be larger at +0.0279 units/year (but did not achieve 0.05 statistical significance).

**Table 1:** Descriptive statistics by DOPPS phase

Variable	Phase 3 April 2006	Phase 4 August 2009	Phase 5 April 2013	Phase 6 August 2017
N	1765	1633	1731	1753
Mean age, years	62.5 (12.5)	64.6 (12.1)	65.2 (12.2)	65.4 (11.8)
Male, %	60%	62%	65%	67%
Mean years on dialysis	8.2 (7.1)	8.3 (7.5)	7.3 (7.6)	7.1 (8)
Mean sp Kt/V	1.35 (0.27)	1.40 (0.28)	1.42 (0.28)	1.49 (0.29)
sp Kt/V ≥ 1.2, %	71%	77%	79%	85%
Mean dialysis treatment duration, minutes	238.2 (30.6)	236.4 (28.4)	238.4 (27.2)	243.3 (26.7)
Mean utrafiltration rate, mL/kg/hr	11.3 (4.1)	8.6 (3.5)	8.7 (3.5)	8.4 (3.3)
Mean blood flow rate, mL/min	198.3 (27)	202.2 (27.5)	207.9 (31.1)	218.4 (35.3)
Blood flow rate ≥ 200 mL/min, %	65%	72%	78%	85%
Hemodiafiltration use, %	6%	6%	8%	23%
Comorbidities, %				
Coronary heart disease	41%	33%	24%	25%
Congestive heart failure	25%	21%	16%	16%
Cerebrovascular disease	13%	15%	11%	17%
Other cardiovascular disease	32%	32%	21%	21%
Peripheral vascular disease	17%	20%	13%	12%
Hypertension	73%	79%	80%	85%
Diabetes	32%	35%	40%	43%
Gastrointestinal bleeding	4%	5%	4%	2%
Neurologic disease	10%	9%	6%	6%
Psychiatric disorder	3%	6%	4%	3%
Recurrent cellulitis	4%	5%	3%	3%
Cancer, non-skin	9%	11%	10%	12%

Shown are mean values (standard deviation) or percent prevalence of the indicated characteristic

**Table 2:** Pre- and post-publication slopes and 95% confidence intervals for linear mixed regression models

Outcome	Pre-Publication slope, per year (95% C.I.^a^)	Post-Publication slope, per year (95% C.I.^a^)	Difference in slopes (95% C.I.^a^)
sp Kt/V	0.0148 (0.0126, 0.0171)	0.0178 (0.0138, 0.0218)	0.0030 (−0.0022, 0.0082)
Treatment Time (min)	0.7008 (0.4654, 0.9361)	1.0809 (0.6968, 1.4651)	0.3802 (−0.1337, 0.8941)
UFR (mL/Kg/hour)	−0.3257 (−0.3548, −0.2965)	0.0106 (−0.0471, 0.0682)	0.3362 (0.2670, 0.4055)
Blood Flow Rate (ml/min)	1.5762 (1.3177, 1.8347)	1.9583 (1.5557, 2.3610)	0.3821 (−0.1610, 0.9252)

*Note*: slope changes were adjusted for covariates as described in Methods

^a^*C.I.* Confidence Interval

**Table 3:** Pre- and post-publication slopes and 95% confidence intervals for logistic mixed regression models

Outcome	Pre-Publication slope, per year (95% C.I.^a^)	Post-Publication slope, per year (95% C.I.^a^)	Difference in slopes (95% C.I.^a^)
Odds of sp Kt/V ≥ 1.2	0.1090 (0.0854, 0.1326)	0.1292 (0.0750, 0.1833)	0.0202 (−0.0434, 0.0837)
Odds of blood flow ≥ 200	0.1191 (0.0970, 0.1412)	0.0682 (0.0297, 0.1067)	−0.0509 (−0.0995, −0.0022)
Odds of hemodiafiltration use	0.0173 (−0.0289, 0.0634)	0.3255 (0.2849, 0.3660)	0.3082 (0.2423, 0.3741)

Note: slope changes were adjusted for covariates as described in Methods

^a^*C.I.* Confidence Interval

The mean blood flow rate increased with time from 198.3 mL/min (April 2006) to 201.7 mL/min (August 2009) and to 202.2 mL/min (April 2013) before publication of the Guidelines, and further increased to 218.4 mL/min after publication of the Guidelines (August 2017, Table 1). Regarding mean blood flow rates over time, the pre-publication slope was +1.5762 mL/min per year compared to the post-publication slope of +1.9583 mL/min per year, with a difference of 0.3821 mL/min per year (95% C.I. [−0.1610, 0.9252]; Table 2).

**Figure 2:**
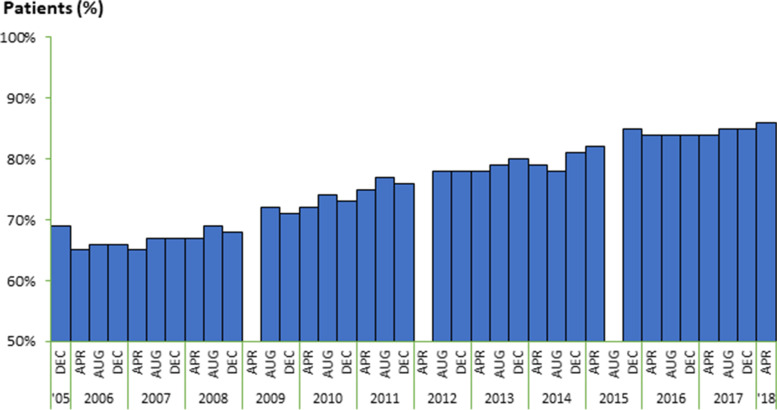
Blood Flow Rate ≥200, mL/min

The percentage of patients with a blood flow rate of ≥200 mL/min increased with time from 65% (April 2006) to 72% (August 2009) and to 78% (April 2013) before publication of the Guidelines, and further increased to 85% after publication of the Guidelines (Figure 2). In logistic models of the odds of patients having a blood flow rate ≥200 mL/min vs. not over time, the pre-publication slope was +0.1191units/year whereas the post-publication slope was 0.0682 units/year. Post/pre slope difference was -0.051 units/year (95% C.I. [−0.0995, −0.0022], Table 3)

The mean treatment time showed very little change over time before publication of the Guidelines (238.4 minutes in April 2006, 236.4 minutes in August 2009, and 238.4 minutes in April 2013), but increased slightly after publication of the Guidelines to 243.3 minutes in August 2017 (Table 1). The mean pre-publication slope for treatment time was 0.7008 minutes/year; the post-publication slope was 1.081 minutes/year, and the post/pre slope difference was 0.3802 minutes/year (95% C.I. [−0.1337, 0.8941], Table 2).

The mean ultrafiltration rate (UFR) decreased with time from 11.3 mL/kg/hr (April 2006) to 8.6 mL/kg/hr (August 2009), rising slightly to 8.7 mL/kg/hr (April 2013) before publication of the Guidelines, and further decreased to 8.4 mL/kg/hr after publication of the Guidelines (August 2017, Table 1). The pre-publication slope for UFR was -0.3257 ml/kg/hr/year, compared to the post-publication slope of 0.0106 mL/kg/hr/year. The post/pre slope difference was 0.3362 ml/kg/hr/year (95% C.I. [0.2670, 0.4055]).

Finally, the percentage of patients receiving HDF was 6%, both in April 2006 and August 2009; which slightly increased to 8% by April 2013 and further increased to 23% by August 2017 (Table 1). In logistic regression mixed models, the pre-publication slope for odds of HDF use was 0.0173 units/year compared to the post-publication slope of 0.3255 units/year. The difference between these two slopes was +0.3082 units/year (95% C.I. [0.2423, 0.3741], Table 3).

## Discussion

Several observational studies have reported that dialysis prescription is associated with mortality [18–20]. These studies reported that the longer the dialysis treatment time, the lower the mortality rates. Furthermore, the HEMO trial found mortality rates to be lower for women when randomized to receive a higher dialysis dose [7, 9]. It has also been reported that patients with a urea reduction rate of ≥65% had a lower mortality rate [21].

The DOPPS, a worldwide observational study, has yielded many significant findings. In particular, the DOPPS has reported that mortality was lower in dialysis patients in Japan than in Europe and the United States ^10 ,11,15,19,20^. The DOPPS has also reported that many Japanese dialysis patients did not reach a single-pool Kt/V value of 1.2, although the KDOQI guidelines recommend this Kt/V value as a minimum to be achieved [6, 10]. The effect of the KDOQI guidelines on Japanese dialysis patients was considered to be less.

In this study, we investigated the effect of the Japanese Guidelines on dialysis prescription in Japanese patients using data from the Japan-DOPPS. We investigated the change in the indices for dialysis prescription from 2005 to 2018 (before and after publication of the Guidelines) in Japan-DOPPS participants. The mean single-pool Kt/V increased with time from 1.35 (April 2006) to 1.40 (August 2009) and to 1.42 (April 2013) before publication of the Guidelines, and further increased to 1.49 after publication of the Guidelines (August 2017). In the linear mixed model, the pre-publication slope for spKt/v was 0.0148 units/month versus 0.0178 units/month for the post-publication slope, thus indicating an increase of 0.003/month (95% C.I. [−0.0022, 0.0082]) in the slope following the Guideline publication. Similarly, in case-mix adjusted analyses of the odds of of patients having an spKt/V ≥1.2 vs. not over time revealed a pre-publication slope of +0.109 units/month (OR 1.12) vs. +0.1292 units/month (OR 1.14) for the post-publication slope, indicating an increase of 0.0202 units/month (95% C.I. [−0.0434, 0.0837) in the slope after the Guideline publication. Although the slope differences before vs. after the Guideline publication date were not statistically significant, a trend towards higher spKt/v achievement after the Guideline publication was observed. The mean percentage of patients with a single pool Kt/V ≥1.2 also increased with time from 71% (April 2006) to 79% (April 2013) before publication of the Guidelines, and to 85% after publication of the Guidelines. These results suggest that the pace of increase in sp Kt/V was accelerated after publication of the Guidelines.

The mean blood flow rate increased with time from 198.3 mL/min (April 2006) to 202.2 mL/min (August 2009) and to 207.9 mL/min (April 2013) before publication of the Guidelines, and further increased to 218.4 mL/min after publication of the Guidelines (August 2017). Mean blood flow rates increased over the entire study period, showing a pre-publication slope of +1.5762 mL/min/mo vs. +1.9583 mL/min/mo for the post-publication slope, indicating a slope increase of 0.3821 (95% C.I. [−0.1610, 0.9252]) after the Guideline publication date. However, case-mix adjusted analyses of the odds of patients having a blood flow rate >200 mL/min vs. not indicated a pre-publication slope of +0.1191 units/year (OR 1.13) compared to a slope of +0.0682 units/year (OR 1.07) after the Guideline publication year. This post- vs. pre-slope difference of −0.0509 units/month was statistically significant, with a 95% C.I. of (−0.0995, −0.0022).

In Japan, the rate of arteriovenous fistula (AVF) use for hemodialysis vascular access is high. An association between higher AVF use and better survival rate has been reported [22–25]. Under the influence of the higher rate of AVF use, the mean blood flow rate in Japanese dialysis patients has been approximately 200 mL/min for a long time [26]. However, the mean increased to 218.4 mL/min in August 2017. The possible reason for this is as follows. In 2012, online HDF was approved for insurance coverage, and the number of patients treated with online HDF increased because of the higher insurance reimbursement price. Patients treated with online HDF tend to have a higher blood flow rate compared with those treated with hemodialysis [27–29].

It is therefore suggested that the increase in the number of patients treated with online HDF beginning in 2012 affected the blood flow rate. In the registry of the Japanese Society for Dialysis Therapy, a similar trend in blood flow rate has been observed [30–32].

Treatment time did not show a marked increase during the period before publication of the Guidelines and showed a slight increase after publication of the Guidelines. The pre-publication slope for treatment time was +0.7008 min/year, the post-publication slope was +1.081 min/year and the post- vs. pre-slope difference was +0.3802. (95% C.I. [−0.1337, 0.8941]). Thus, an increasing trend towards slightly longer treatment time was observed. The DOPPS has reported that treatment time was longer in hemodialysis patients in Japan than in the United States [19]. It is also reported that every 30-minute longer treatment time in an HD facility was associated with a 6% lower adjusted risk for death [19]. Despite the fact that dialysis patients are generally reluctant to extend their treatment time, the trend per year significantly increased after publication of the Guidelines, suggesting that the Guidelines had a significant influence on the same.

There were no marked changes in the UFR, which is an important index for determining fluid removal. The UFR in the present study was relatively low, 11.3 mL/kg/hr in 2009 and 8.6 mL/kg/hr in 2013, and thereafter decreasing slightly to 8.4 mL/kg/hr in 2017. The Guidelines recommend that UFR be 15 mL/kg/hr or lower. The DOPPS reported that UFR ≤10 mL/kg/hr is associated with better survival [18]. Movilli et al. also recommended that UFR be ≤12 mL/kg/hr [33] and Flythe et al. recommended <13 mL/kg/hr [34].

Recently, Wong et al reported trends towards lower UFR levels and lower interdialytic weight gain in the US, Canada, Europe, and Australia/New Zealand based on DOPPS data from 2002-2014 [35]. In the “Japanese Society for Dialysis Therapy Guidelines for Management of Cardiovascular Diseases in Patients on Chronic Hemodialysis,” it is recommended that the amount of water removed per unit time should be mitigated to avoid a drop in blood pressure during dialysis, and prolongation of the duration of dialysis should be considered [36]. Although the UFR in Japanese dialysis patients had been low, it further decreased to 8.4 mL/kg/hr after publication of the Guidelines, suggesting an influence of the Guidelines.

The percent of patients treated with HDF was 6% in both April 2006 and August 2009; the value slightly increased to 8% in April 2013 and further increased to 23% in August 2017. The pre-publication slope for odds of patients prescribed HDF was +0.0173 units/year (OR 1.02) vs. the post-publication slope of +0.3255 units/year (OR 1.38). Thus the slope in odds of HDF use markedly increased by +0.3082 units/year (95% C.I. [0.2423, 0.3741]) following publication of the Guidelines. As mentioned earlier, the plausible reason for this is that online HDF was approved for insurance coverage in 2012. However, the percentage of patients treated with HDF only increased to 8% in April 2013 despite online HDF being added to the insurance coverage list in 2012. This might be due to the fact that the dialysis console for online HDF was still not in widespread use at that time due to more limited availability. The rapid increase in the percentage of patients treated with online HDF in 2017 was probably due to the much greater availability of this dialysis console in Japan by 2017.

Online haemodiafiltration (HDF) today represents the most advanced and innovative form of renal replacement therapy, and online HDF treatment is now widespread in many countries. There are several characteristics to note regarding Japanese online HDF. The first is the high use of the pre-dilution mode; the second is treatment at a relatively low blood flow rate compared with Europe; and the third is the typical use of a high performance membrane filter. These three characteristics are mutually related. The reason for the wide spread use of pre-dilution mode in online HDF is due to the low blood flow rate utilized in Japan. Furthermore, pre-dilution mode online HDF has the advantage of reducing albumin loss when using a high-performance membrane filter.

In Japan, online HDF is selected as the most advanced blood purification modality to eliminate uremic solutes similar in molecular weight to α1–macroglobulin, but not for removing β2-microglobulin. In JSDT Renal Registry data (2017), the mean removal rate was 60.7% for HD patients overall and 71.4% for HDF patients overall, indicating that HDF patients overall had a higher mean value than HD patients overall. Nevertheless, there was no marked difference in the pre-dialysis β2-microglobulin levels between HD and online HDF patient groups [32].

Roumelioti et al [37] have also reported that various factors such as a blood purification mode, dialytic fluid flow rate, blood flow rate, and membrane materials of filters or dialyzers influence clearance of the β2-microglobulin. In Japan, many kinds of dialyzers or filters of various membrane materials are used [32]; this factor may influence blood concentration levels of β2-microglobulin.

However, removing solutes similar in size to α1-microglobulin results in greater loss of serum albumin. It is thought that uremic solutes around α1–microglobulin in size can be safely and effectively removed while reducing albumin leak by using the pre-dilution mode when performing online HDF. In the technical aspects, the lower blood flow rate used in Japan compared with that used in Europe may be suitable for pre-dilution mode online HDF using a high performance membrane filter in Japan [29–32, 38–40].

On the basis of these various factors (incentive in health insurance reimbursement, widespread availability of an HDF console machine, recommendations in the Guidelines, expectations for advanced modality, suitability in the technical aspects), the number of “online HDF” cases increased rapidly from 2013 – 2018.

Our study has several limitations, including its observational design. We investigated the impact of the Guidelines on dialysis prescription, but we could not exclude the influences of the following confounding factors. First, we could not eliminate the influence of the revision in the medical payment system. As is well known, there is a possibility that the selection of treatment modality is greatly influenced by insurance coverage. The impact of this factor might have been as great as that of the Guidelines. In addition, the blood flow rate might have been influenced by changes in treatment mode, which could be a confounding factor. Lastly, even though patients and facilities included in JDOPPS were randomly selected for participation, we cannot entirely exclude patient selection bias depending upon which facilities and patients consented to study participation. This can affect the generalizability of study findings to the extent of such a possible bias existing.

## Conclusions

This study investigated the effect of the Japanese Society for Dialysis Therapy Clinical Guideline for “Maintenance Hemodialysis: Hemodialysis Prescriptions” published in 2013 on hemodialysis prescription in Japan using the data obtained from Japan-DOPPS. The results showed increases in several parameters for dialysis therapy including single-pool Kt/V, blood flow rate, treatment time, and HDF use after publication of the Guidelines, suggesting an influence of the Guidelines on dialysis prescription.

## Data Availability

The datasets used and/or analysed during the current study are available from the corresponding author on reasonable request.

## Abbreviations

AIC: Akaike information criterion
AVF: Arteriovenous fistula
BIC: Bayesian information criterion
BFR: Blood flow rate
CV: Cardiovascular
CVA: Cerebrovascular accident
DOPPS: Dialysis Outcomes and Practice Patterns Study
GI: Gastrointestinal
GEE: Generalized estimating equation
GL: Guidelines
HDF: Hemodiafiltration
HD: Hemodialysis
KDOQI: Kidney Disease Outcomes Quality Initiative
spKt/V: Single-pool Kt/V
TT: Treatment time
UFR: Ultrafiltration rate
